# Supplementary material for: Early Astrocytic Dysfunction Is Associated with Mistuned Synapses as well as Anxiety and Depressive-Like Behavior in the AppNL-F Mouse Model of Alzheimer’s Disease
Source: J Alzheimers Dis. 2024 Jul 30;100(3):1017–37. doi: 10.3233/JAD-231461 (PMC11307019; doi:10.3233/JAD-231461)
Supplement: Supplementary Material [file jad-100-jad231461-s001.pdf]

Supplementary Material

Early Astrocytic Dysfunction Is Associated with Mistuned Synapses as well as Anxiety and Depressive-Like Behavior in the AppNL-F Mouse Model of Alzheimer's Disease

SUPPLEMENTARY METHODS

Amyloid-beta (Aβ) ELISA

Perfusion and brain extraction

Mice were anesthetized with a mixture of ketamine (100 mg/kg) and xylazine (16 mg/kg) administered via i.p route. In absence of corneal reflexes, mice were transcardially perfused with NaCl 0.9%. The brain was extracted, and hippocampi, cortex, and cerebellum were dissected in dry ice and kept at -80°C until further use.

Brain homogenization

The brain regions were homogenized at 1:5 weight/volume ratio in precellys homogenization tubes (Bertin Technologies, P000933-LYSK0-A). A three-step homogenization was applied: Tris-buffered saline (TBS) to isolate soluble Aβ, TBS with 1% triton-x100 (TBS-T; Sigma-Aldrich, X100) for isolation of membrane-associated Aβ, and 70% (v/v) formic acid (FA; Sigma-Aldrich, F0507) for the insoluble Aβ fraction. TBS-homogenized brains were centrifuged at 16 000 x g for 60 min at +4°C, the supernatant was collected. The pellets were subsequently homogenized in TBS-T and centrifuged at 16 000 x g for 60 min at +4°C and supernatant was collected. The pellets were dissolved in 70% (v/v) FA, centrifuged at 16 000 x g for 60 min at +4°C and the supernatant was collected and immediately neutralized with 1M Tris pH 8.0 (Thermo Fisher Scientific, AM9856).

Aβ immunostaining

The brains were sliced in 40 μm thick sections using a cryostat (Thermo Scientific Cryostar NX70). Free-floating slices were treated with a 10% formic acid solution for 10 min for epitope retrieval. After blocking with normal donkey serum, slices were incubated with primary Aβ antibody (Covence Signet SZG-39300-200; 1:5000) overnight at 4°C and then with Alexa fluor 647 (1:5000; Invitrogen A31571) for 2 h. DAPI (1:5000) was added to counterstain nuclei. Slices

were then mounted on non-coated microscope glass coverslips using the fluoromount mounting medium (Invitrogen, 00-4958-02). Images were taken on a confocal microscope (Zeiss LSM700) with a 10x objective.

Aβ ELISA

The three Aβ fractions were analyzed using a homogenous sandwich ELISA using the Aβ N-terminal specific antibody 3D6 (murine version of Bapineuzumab, produced in-house) as both capture and detection antibody. 96-wells ELISA plates were coated with 1 μg/mL 3D6 and incubated over night at +4°C. Plates were blocked with 1% (w/v) BSA in PBS 1X for 2 h. Dilutions of TBS and TBS-T brain fractions (1:10 (v/v)) and FA brain fraction (1:200 (v/v)) in ELISA incubation buffer (EIB; 0.05% tween-20 (Sigma-Aldrich, P9416), 0.1% w/v BSA in PBS 1X) were added to the ELISA plate and incubated over nigh at +4°C. Biotinylated 3D6 (prepared in-house; 1 μg/mL in EIB) was incubated for 2 h at room temperature followed by 1 h of streptavidin-HRP (Mabtech, 3310-9-1000) at room temperature. The ELISA was developed with K-blue Aqueous TMB substrate (Neogen Corp., 331177) and read with a spectrophotometer (Tecan, Spark) at 450 nm.

Astrocyte density

Astrocyte density was manually evaluated by an experimenter blinded to experimental condition, using the software Image J. Briefly, GFAP-positive cells were counted in Z-projected images. Two images per animal were obtained using confocal imaging. DAPI was used to determine the centroid and to facilitate the identification of GFAP positive cells. Cells with putative nuclei outside of the field were discarded. The density was calculated as follow: $\frac{\text{Number of cells}}{\text{Size of image } (\mu m^2)}$.

Behavioral analysis

Splash test

The animals were isolated in a new cage and habituated for at least 20 min. 200 μL of 10% sucrose solution was squirted on the mouse's snout and the grooming behavior (latency to first, frequency and total time), used to evaluate self-care, was manually scored for 5 min. Mice were placed back together in their home cage after the experiment.

Sucrose preference test

Each animal was singled housed and habituated to drink water from two identical bottles for 48 h. During the following 72 h, mice could choose between water and a 1% sucrose solution. Sucrose solution intake was measured by weighing the bottles before and after the last 24 h and expressed as the percentage of the total amount of liquid ingested, normalized to the mouse body weight. Each bottle was replaced and switched from left to right daily.

Rotarod test

Animals were tested on a rotating bar of 5 cm diameter on which mice were placed facing the sense of the rotation. Animals were habituated to the device for 30 s at a constant speed of 5 rotations per minute (rpm) the day before the experiment. This was followed by 3 trials per animal with 15 min between each trial. During the trials, mice were placed on an accelerating rod increasing from 5 to 50 rpm for 5 min. The test is stopped when the mouse fall from the rod. The latency before falling and the maximum rotation speed before falling are recorded.

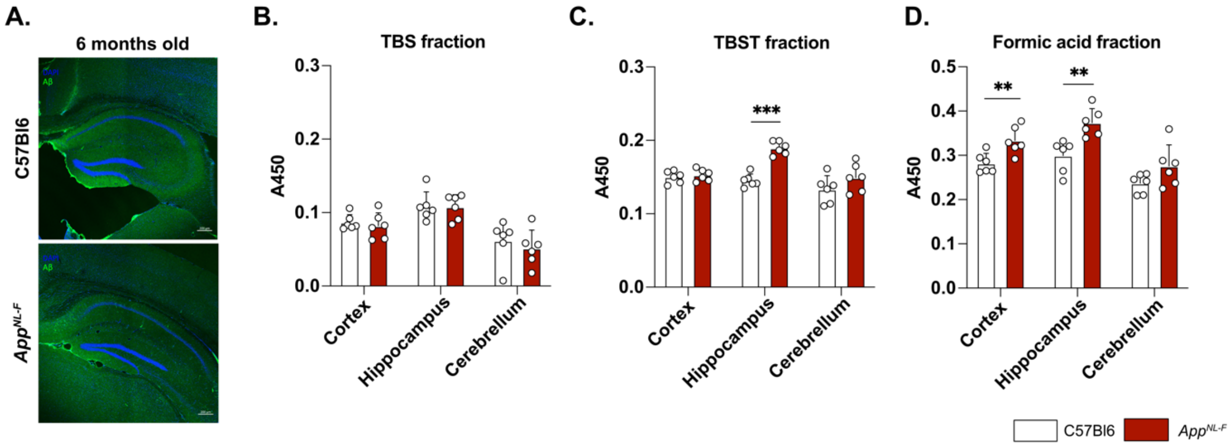

Supplementary Figure 1. ***App***$^{NL-F}$ **mice do not display Aβ plaques but higher levels of Aβ in the insoluble fraction in the hippocampus.** Immunolabelling of Aβ (A) reveals the absence of plaques in the hippocampus of 6-month-old *App*$^{NL-F}$ mice, compared to C57Bl6 mice. ELISA analysis of different Aβ fractions shows unchanged concentrations in non-membrane-bound Aβ (TBS fraction, (B) in the hippocampus, the cortex and the cerebellum. However, increased concentration of membrane-bound Aβ (TBST fraction) in the hippocampus (C) but not in the cortex nor the cerebellum were observed. Our analysis also revealed an increased formic acid fraction (i.e., non-soluble dimers) in both the cortex and the hippocampus (D). For the three fractions: Two-way ANOVA with the genotype and the brain region as the main factors. Bonferroni multiple comparison: **$p<0.01$; ***$p<0.001$ statistically significant as shown. C57Bl6: n=6, *App*$^{NL-F}$: n=6. Panel A: scale bar 200m.

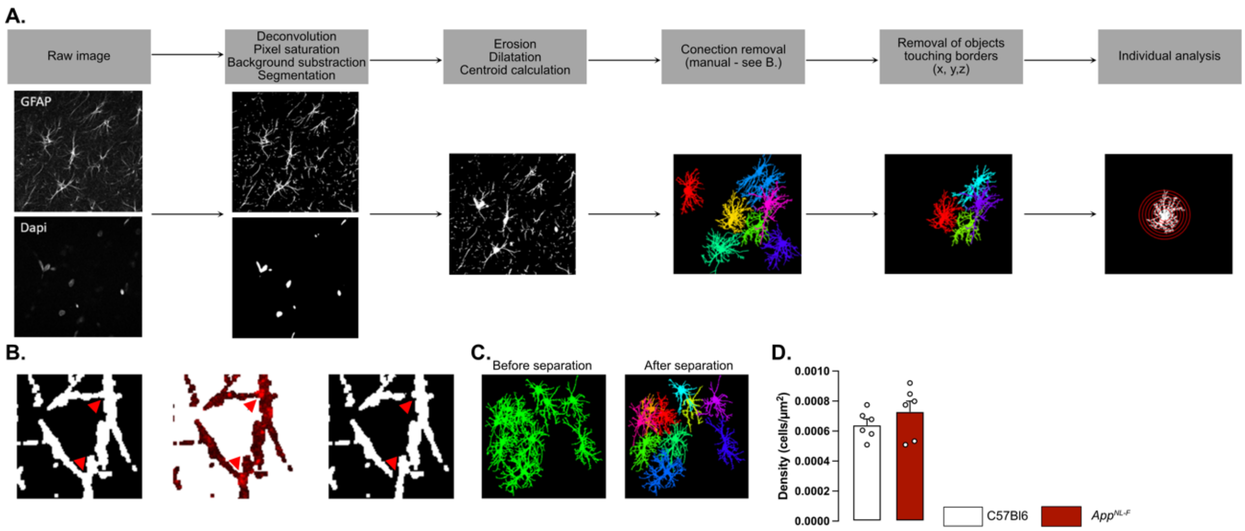

Supplementary Figure 2. Astrocyte morphology analysis (*related to Fig. 1*). Using Z-stack images of GFAP and DAPI staining were merged in a z-projection and were treated according to the presented workflow (A). Before reconstruction astrocytes were manually separated (B): the first image shows the connection (red arrows) on the deconvoluted image while the second image shows the raw image of the connexin 43 (Cx43) staining, use to discriminate connecting hubs between astrocytes. The third image shows the absence of connections after manual removal. (C) depicts how the syncytium looks like before, and after separation. Once all astrocytes have been separated, the morphology of each cell was 3D reconstructed and individually analyzed. (D) shows the number of astrocytes per image, calculated on obtained z-projections (two images per mouse, three mice per group).

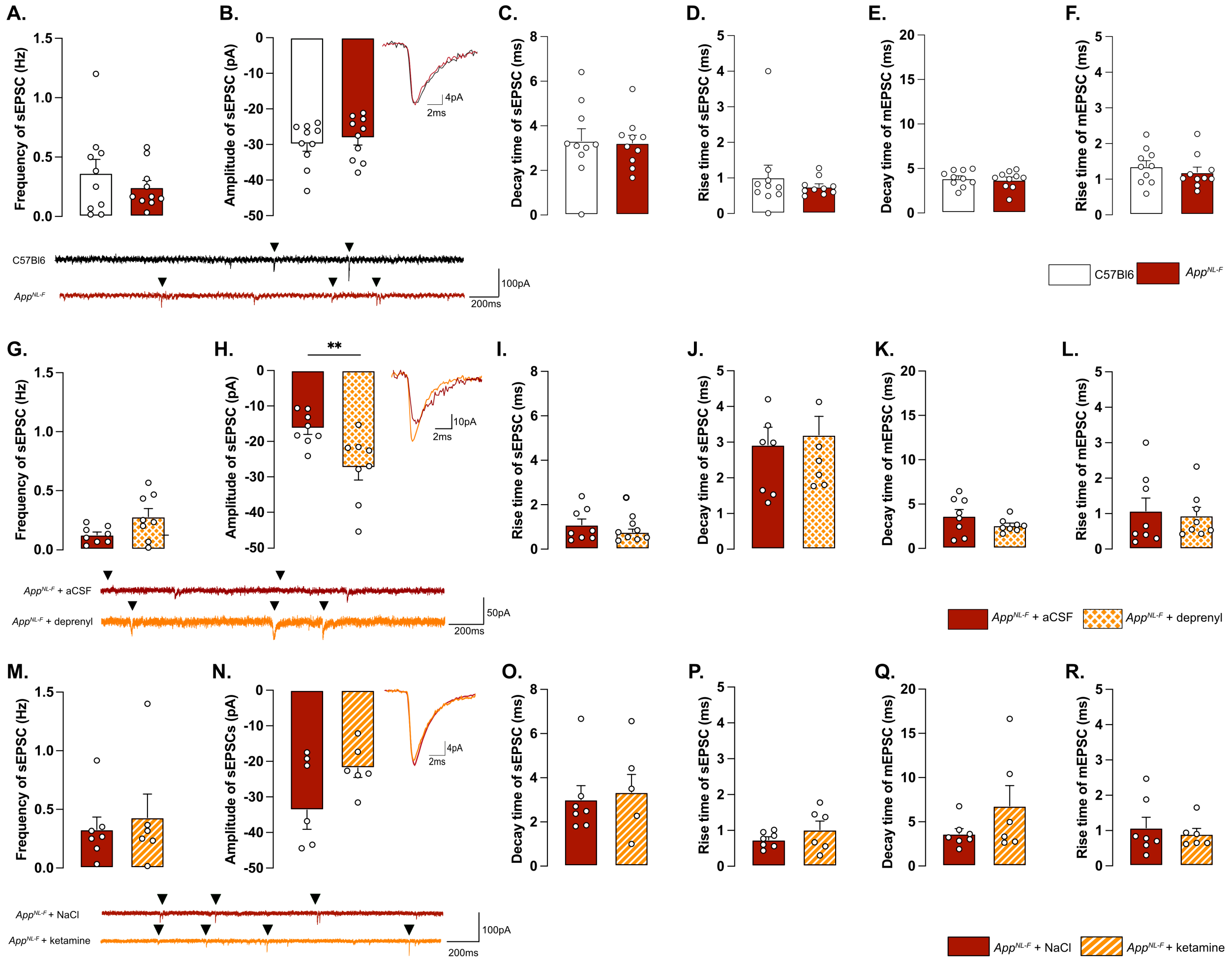

Supplementary Figure 3. Complementary electrophysiological measurements (*related to Fig. 2, 3, and 7*). Patch clamp experiments revealed no difference in frequency (A), amplitude (B) decay time (C), or rise time (D) of sEPSC. No differences were unveiled either in decay time (E) nor rise time (F) of mEPSC. Representative traces for sEPSC are depicted under the graphs. C57Bl6 controls: n=10 cells recorded in seven animals, *App*$^{NL\text{-}F}$: n=10 cells recorded in seven animals. Pre-treatment with deprenyl did not affect the frequency of sEPSC (G) but increased the amplitude (H) Representative traces are depicted under the graphs. Neither the rise time or the decay time for sEPSC (I and J, respectively) and mEPSC (K and L, respectively) were affected by deprenyl. *App*$^{NL\text{-}F}$ + aCSF: n=8 cells recorded in four animals; *App*$^{NL\text{-}F}$ + deprenyl: n=8 cells recorded in four animals. No differences were unveiled in frequency (M), amplitude (N), decay time (O) or rise time (P) of sEPSC after ketamine treatment. The NMDA receptor blocker did not affect the decay time (Q) nor the rise time (R) of mEPSC. Representative traces for sEPSC are depicted under the graphs. *App*$^{NL\text{-}F}$ + NaCl: n=7 cells recorded in five animals, *App*$^{NL\text{-}F}$ + ketamine: n=6 cells recorded in six animals.

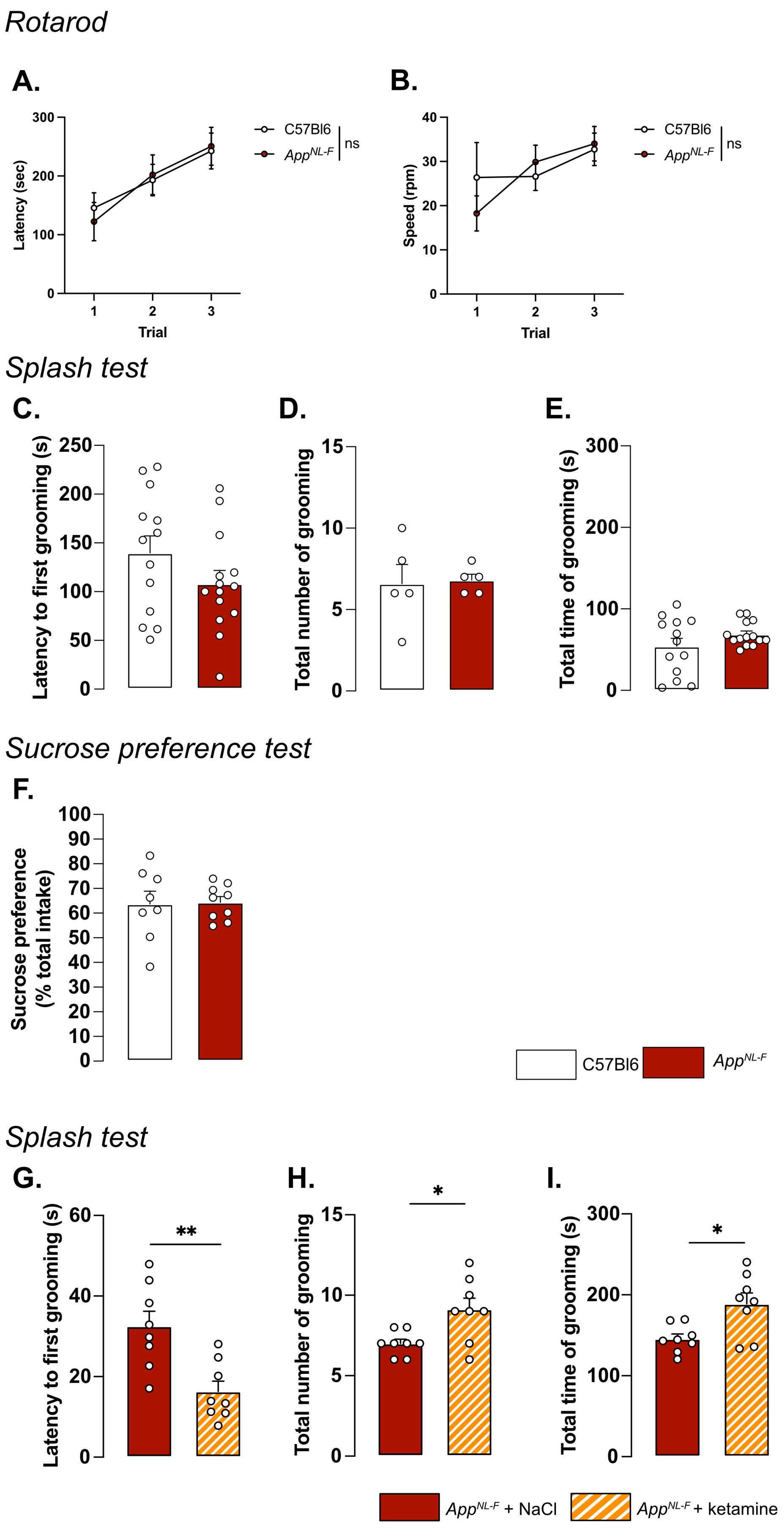
Rotarod
A.
Latency (sec)
Trial
C57Bl6
App^NL-F
ns
B.
Speed (rpm)
Trial
C57Bl6
App^NL-F
ns
Splash test
C.
Latency to first grooming (s)
D.
Total number of grooming
E.
Total time of grooming (s)
Sucrose preference test
F.
Sucrose preference (% total intake)
C57Bl6
App^NL-F
Splash test
G.
Latency to first grooming (s)
**
H.
Total number of grooming
*
I.
Total time of grooming (s)
*
App^NL-F + NaCl
App^NL-F + ketamine

Supplementary Figure 4. Rotarod, splash test and sucrose preference test (*related to Figs. 5 and 6*). In the rotarod, no differences were unveiled in the latency to fall from the rotarod (A) nor in the maximum speed before falling (B). C57Bl6: n=8, *App*$^{NL\text{-}F}$: n=8. Bonferroni multiple comparison. In the splash test, no differences were unveiled in the latency to first grooming (C), total number of grooming (D) nor in the total time of grooming (E). C57Bl6: n=13, *App*$^{NL\text{-}F}$: n=14. For number of groomings, n=4 and n=5 for C57Bl6 and *App*$^{NL\text{-}F}$ respectively since it was only recorded in one of two experiments. In the sucrose preference test, no difference was unveiled in the sucrose consumption (F) C57Bl6: n=8, *App*$^{NL\text{-}F}$: n=9. After a single injection of ketamine (5mg/kg), the latency to first grooming (G), total number of grooming (H) and in the total time of grooming (I) were increased, compared to saline treated animals. Student t-test: *$p<0.05$, **$p<0.01$ statistically different as shown. *App*$^{NL\text{-}F}$ + NaCl: n=8, *App*$^{NL\text{-}F}$ + ketamine: n=8.

		zSc_param													zSc_test					
Cohort	Genotype	zSC_OF_Distance	zSC_OF_Center entries	zSC_OF_Center time	zSC_OF_Center/Total distance ratio	zSC_EPM_Open arms entries	zSC_EPM_Open arms time	zSC_EPM_Open/Total arms entries	zSC_ST__Grooming latency	zSC_ST_Grooming frequency	zSC_ST_Grooming duration	zSC_TST_Immobility latency	zSC_TST_Immobility duration	zSC_SPT_Sucrose consumption	zSC_OF	zSC_EPM	zSC_ST	zSC_TST	zSC_SPT	Emotionality z-score
Formula		*(X-μ)/σ*													*Average parameter (per test)*					*Average z-score*
1	C57Bl6	0.49	0.42	-1.88	1.59	0.50	-0.26	0.57				-0.64	-1.73		0.15	0.27		-1.19		-0.25
1	C57Bl6	0.95	0.60	-0.85	1.32	-2.18	-1.55	-1.53				1.84	0.17		0.51	-1.75		1.00		-0.08
1	C57Bl6	1.56	1.59	1.03	0.34	-0.17	-0.79	-0.47				-0.20	-0.48		1.13	-0.48		-0.34		0.10
1	C57Bl6	-1.16	-0.83	0.58	-1.03	-0.17	-0.26	-0.64				-1.00	0.27		-0.61	-0.35		-0.37		-0.44
1	C57Bl6	0.22	0.42	0.48	-0.31	1.18	1.57	1.35				-0.49	-0.55		0.20	1.37		-0.52		0.35
1	C57Bl6	-0.37	0.25	1.02	-0.97	-0.17	-0.26	-0.84				0.82	0.37		-0.02	-0.42		0.59		0.05
1	C57Bl6	-1.33	-1.27	-0.21	-0.68	0.50	0.50	0.77				0.60	1.79		-0.87	0.59		1.20		0.31
1	C57Bl6	-0.37	-1.18	-0.16	-0.25	0.50	1.03	0.77				-0.93	0.17		-0.49	0.77		-0.38		-0.03
2	C57Bl6								-0.24		-0.80	-0.16	0.40	0.17			-0.52	0.12	0.17	-0.08
2	C57Bl6								1.05		-0.49	-0.69	0.38	0.85			0.28	-0.15	0.85	0.33
2	C57Bl6								0.74		1.09	-0.87	0.53	-1.74			0.92	-0.17	-1.74	-0.33
2	C57Bl6								0.17		0.48	0.76	-0.32	1.34			0.33	0.22	1.34	0.63
2	C57Bl6								-1.01		-1.00	-1.17	1.57	-0.91			-1.01	0.20	-0.91	-0.57
2	C57Bl6								0.10		-0.97	0.89	-1.27	-0.24			-0.43	-0.19	-0.24	-0.29
2	C57Bl6								0.99		0.03	1.67	-1.48	-0.17			0.51	0.09	-0.17	0.14
2	C57Bl6								-1.80		1.66	-0.44	0.19	0.69			-0.07	-0.13	0.69	0.16
3	C57Bl6								-0.98	1.38	-1.63						-0.41			-0.41
3	C57Bl6								-1.26	-1.30	0.35						-0.74			-0.74
3	C57Bl6								-0.48	0.23	0.57						0.11			0.11
3	C57Bl6								0.05	0.23	-0.21						0.02			0.02
3	C57Bl6								-1.56	-0.54	0.92						-0.39			-0.39
1	App^{NL-F}	1.79	1.67	2.18	-0.60	1.18	1.57	1.35				-2.32	-0.34		1.26	1.37		-1.33		0.43
1	App^{NL-F}	1.43	0.60	0.63	0.57	-0.17	-0.26	-0.21				-2.10	1.89		0.81	-0.21		-0.10		0.16
1	App^{NL-F}	1.25	1.05	1.13	-0.06	-0.17	0.28	0.06				0.53	0.33		0.84	0.06		0.43		0.44
1	App^{NL-F}	2.32	1.59	2.08	0.07	0.50	0.28	0.14				-0.57	0.91		1.51	0.31		0.17		0.66
1	App^{NL-F}	1.97	1.41	-6.53	9.29	-0.84	-1.76	-0.99				-1.37	1.93		1.53	-1.20		0.28		0.21
1	App^{NL-F}	1.45	1.05	-0.16	1.34	-0.17	-0.04	-1.08				0.09	0.57		0.92	-0.43		0.33		0.27
1	App^{NL-F}	1.83	1.41	0.21	1.53	0.50	0.93	-0.21				-2.46	2.60		1.24	0.41		0.07		0.57
1	App^{NL-F}	2.21	1.41	-0.06	2.55	1.18	1.57	1.35				-0.64	0.81		1.53	1.37		0.09		0.99
2	App^{NL-F}								-1.02		0.56	-0.89	4.11	0.24			-0.23	1.61	0.24	0.54
2	App^{NL-F}								-0.16		0.19	-0.55	1.70	0.70			0.02	0.57	0.70	0.43
2	App^{NL-F}								0.67		0.69	-1.23	8.42	0.58			0.68	3.60	0.58	1.62
2	App^{NL-F}								-1.16		0.52	-0.75	0.40	-0.34			-0.32	-0.17	-0.34	-0.28
2	App^{NL-F}								-1.67		0.62	-0.52	0.98	-0.61			-0.52	0.23	-0.61	-0.30
2	App^{NL-F}								0.45		0.34	-1.08	5.44	0.17			0.39	2.18	0.17	0.92
2	App^{NL-F}								-1.54		1.37	-0.74	1.58	-0.24			-0.08	0.42	-0.24	0.03
2	App^{NL-F}								-0.89		1.37	-0.27	3.91	0.39			0.24	1.82	0.39	0.82
2	App^{NL-F}								-1.16		0.34	-0.80	3.77	-0.52			-0.41	1.49	-0.52	0.18
3	App^{NL-F}								-1.09	-0.54							-0.74			-0.74
3	App^{NL-F}								-0.84	0.23							-0.03			-0.03
3	App^{NL-F}								-0.61	-0.15							-0.38			-0.38
3	App^{NL-F}								-1.67	-0.15							-0.40			-0.40

Supplementary Table 1. Calculation of the emotionality z-score (*related to Fig. 5*). For each test, a z-score ($z = (X - \mu)/\sigma$) is calculated for each parameter of consideration (zSC_param). All the zSC-parameters are then averaged per test leading to a test z-score (zSc_test). The average of each score gives the final z-score per animal. X: individual values for the considered parameter; μ: mean of the control group; σ: standard deviation of the control group. In this figure, C57Bl6 mice have been used as the control group.

		zSc_param												zSc_test				
Cohort	Treatment	zSC_OF_Distance	zSC_OF_Center entries	zSC_OF_Center time	zSC_OF_Center/Total distance ratio	zSC_EPM_Open arms entries	zSC_EPM_Open arms time	zSC_EPM_Open/Total arms entries	zSC_ST__Grooming latency	zSC_ST_Grooming frequency	zSC_ST_Grooming duration	zSC_TST_Immobility latency	zSC_TST_Immobility duration	zSC_OF	zSC_EPM	zSC_ST	zSC_TST	Emotionality z-score
Formula		*(X-μ)/σ*												*Average parameter (per test)*				*Average z-score*
1	*App*$^{NL-F}$ + NaCl	0.61	0.45	0.33	0.21							1.59	-0.22	0.40			0.68	0.54
1	*App*$^{NL-F}$ + NaCl	1.37	0.02	-0.52	-1.35							0.42	0.92	-0.11			0.67	0.27
1	*App*$^{NL-F}$ + NaCl	0.29	0.56	0.25	0.30							-1.06	0.77	0.35			-0.14	0.10
1	*App*$^{NL-F}$ + NaCl	1.27	1.32	1.61	1.29							0.95	-1.63	1.37			-0.33	0.51
1	*App*$^{NL-F}$ + NaCl	-0.23	0.23	-1.87	-1.29							-0.42	-0.48	-0.79			-0.45	-0.62
1	*App*$^{NL-F}$ + NaCl	-0.26	0.45	-0.27	0.11							0.42	-0.87	0.01			-0.22	-0.10
1	*App*$^{NL-F}$ + NaCl	1.48	0.88	1.15	0.63							-1.06	0.18	1.04			-0.43	0.30
1	*App*$^{NL-F}$ + NaCl	0.41	-0.19	-1.21	-1.20							-0.85	1.33	-0.54			0.24	-0.15
2	*App*$^{NL-F}$ + NaCl	-0.50	0.23	1.23	1.48	-0.99	-1.78	-1.52	0.03	-1.32	1.32			0.61	-1.43	0.01		-0.27
2	*App*$^{NL-F}$ + NaCl	-0.12	0.67	0.88	1.06	0.77	0.44	0.79	-0.94	-1.32	1.39			0.62	0.67	-0.28		0.33
2	*App*$^{NL-F}$ + NaCl	1.60	1.21	1.58	1.11	-0.11	-2.28	-0.25	-0.26	1.32	-0.06			1.38	-0.88	0.33		0.27
2	*App*$^{NL-F}$ + NaCl	0.66	-0.19	-0.97	-1.17	0.77	0.44	0.79	1.09	0	-0.32			-0.42	0.67	0.25		0.16
2	*App*$^{NL-F}$ + NaCl	-0.60	-1.06	-0.26	0.27	-0.11	-1.93	0.04	0.55	0	-0.95			-0.41	-0.66	-0.13		-0.40
2	*App*$^{NL-F}$ + NaCl	-1.13	-2.47	-1.52	-0.44	0.77	0.44	0.79	-1.47	0	-1.47			-1.39	0.67	-0.98		-0.56
2	*App*$^{NL-F}$ + NaCl	-0.36	-0.84	-1.92	-1.24	0.77	0.44	0.79	1.46	1.32	0.24			-1.09	0.67	1.01		0.19
2	*App*$^{NL-F}$ + NaCl	-1.19	-1.28	-0.70	0.18	-1.88	-1.70	-1.44	-0.46	0	-0.14			-0.74	-1.67	-0.20		-0.87
1	*App*$^{NL-F}$ + ketamine	0.41	-0.41	-4.72	-4.66							1.27	-0.45	-2.34			0.40	-0.96
1	*App*$^{NL-F}$ + ketamine	-1.85	-2.14	-1.65	-0.20							-2.66	2.57	-1.46			-0.04	-0.75
1	*App*$^{NL-F}$ + ketamine	-0.08	0.56	0.35	0.58							0.85	1.01	0.35			0.93	0.64
1	*App*$^{NL-F}$ + ketamine	-1.26	-1.71	-1.44	-0.32							1.38	0.86	-1.18			1.12	-0.03
1	*App*$^{NL-F}$ + ketamine	-0.94	-1.06	-1.16	-0.27							-3.29	1.21	-0.86			-1.04	-0.95
1	*App*$^{NL-F}$ + ketamine	0.33	-0.63	-2.18	-2.08							-2.76	1.39	-1.13			-0.68	-0.91
1	*App*$^{NL-F}$ + ketamine	0.60	0.02	-1.06	-1.20							-2.34	1.42	-0.41			-0.45	-0.43
1	*App*$^{NL-F}$ + ketamine	-0.29	-0.63	-0.35	0.05							-0.74	1.98	-0.30			0.61	0.15
2	*App*$^{NL-F}$ + ketamine	3.42	1.75	-0.76	-6.51	-0.11	0.16	-0.94	-2.35	2.64	5.55			-0.52	-0.29	1.94		0.37
2	*App*$^{NL-F}$ + ketamine	1.43	0.67	0.83	0.26	-0.99	-4.40	-1.52	-1.17	2.64	4.68			0.79	-2.30	2.05		0.18
2	*App*$^{NL-F}$ + ketamine	0.64	-0.30	0.63	0.50	-0.11	0.27	-0.69	-1.77	2.64	2.96			0.36	-0.17	1.28		0.49
2	*App*$^{NL-F}$ + ketamine	-0.13	-0.08	0.50	0.74	-0.99	-1.56	-1.52	-1.82	-1.32	-0.70			0.25	-1.36	-1.28		-0.79
2	*App*$^{NL-F}$ + ketamine	1.75	1.21	2.04	1.67	-0.99	-3.68	-1.29	-2.06	5.29	3.55			1.67	-1.99	2.26		0.64
2	*App*$^{NL-F}$ + ketamine	0.46	-0.19	-0.37	-0.40	0.77	0.44	0.79	-0.42	0	-0.57			-0.12	0.67	-0.33		0.07
2	*App*$^{NL-F}$ + ketamine	1.09	1.10	0.75	0.39	-0.11	0.02	-0.07	-0.73	3.96	2.04			0.83	-0.05	1.75		0.84
2	*App*$^{NL-F}$ + ketamine	0.69	0.45	-2.91	-3.21	-0.11	-0.26	-0.36	-2.02	6.61	2.70			-1.24	-0.24	2.43		0.31

Supplementary Table 2. Calculation of the emotionality z-Score (*related to Fig. 6*). For each paradigm, a z-score ($z = (X - \mu)/\sigma$) is calculated for each parameter of consideration (zSC_param). All the zSC-parameters are then averaged per test leading to a test z-score (zSc_test). The average of each score gives the final z-score per animal. X: individual values for the considered parameter; μ: mean of the control group; σ: standard deviation of the control group. In this figure, *App*$^{NL-F}$ + NaCl mice have been used as the control group.
